# Reconstruction of Long Anterior Urethral Strictures by Dorsally Quilted Penile Skin Flap

**DOI:** 10.5402/2012/651513

**Published:** 2012-03-04

**Authors:** Abdel Moneim M. Abuzeid, M. S. Abdel Kader

**Affiliations:** ^1^Urology Department, Sohag Faculty of Medicine, Sohag University, Sohag 82524, Egypt; ^2^Urology Department, Qena Faculty of Medicine, South Valley University, Qena 83523, Egypt

## Abstract

*Objective*. To present the results of reconstruction of long (>5 cm), penile, bulbar, and bulbopenile urethral strictures by penile skin flap as dorsal onlay in one-stage procedure. *Patients and Methods*. Between January, 1998 and December, 2004, 18 patients (aged from 28-65 years) presented with long urethral strictures, 5.6–13.2 cm (penile in 6, bulbar in 2 and combined in 10 cases), those were repaired utilizing long penile skin flaps placed as dorsal onlay flap in one stage (Orandi flap 6 cm in 6 cases, circular flaps 7–10 cm in 8, and spiral flaps 10–15 cm in 4). Followup of all patients after reconstruction included urine flow rate at weekly intervals, RUG at 6–12 weeks, and urethrocystoscopy at 12 and 18 months. *Results*. The urethral patency was achieved in 77% of patients. The complications were fistula in one patient (5.5%), restricture occurred in 3 patients (16.6%) that required visual internal urethrotomy and two patients (11%) showed curvature on erection that dose not interfere with sexual intercourse. Diverticulum (penile urethra) was seen in one patient (5.5%) containing stones and was excised surgically. There was penile skin loss in 3 patients (16.6%). All patients completed at least one-year followup period. *Conclusion*. Free penile skin flaps offer good results (functional and cosmetic) in long penile and/or bulbar urethral strictures. Meticulously fashioned longitudinal, circular or spiral penile skin flaps could bridge urethral defects up to 15 cm long.

## 1. Introduction

 No single approach is appropriate for all urethral strictures. For surgical repairs, proper procedure selection and surgical expertise are of paramount importance [1]. The reconstructive surgeon should be fully familiar with the use of both flaps and grafts to deal with any condition of the urethra at time of surgery.

 Controversies exist over the best means of reconstructing the anterior urethra [2]. Penile skin flaps, which have ample vascular pedicle, were considered the most reliable material for reconstruction of long or complex strictures [3]. Dorsal placement of penile skin flaps and free grafts has yielded superior outcomes compared to ventral placement [4].

 The objective of this prospective study is to present the results of reconstruction of long (>5 cm), penile, bulbar, and bulbopenile urethral strictures by penile skin flap as dorsal onlay in one-stage procedure.

## 2. Patients and Methods

 During the period from January, 1998 to December, 2004, 18 patients had long penile, bulbar, or bulbopenile strictures repaired utilizing long penile skin flaps placed as dorsal onlay flap in one stage. All patients underwent routine laboratory investigations (e.g., complete blood count, blood urea and serum creatinine, bleeding profile, and random blood sugar), upper tract evaluation by US and Intravenous urography, uroflowmetry (the mean peak flow rate was 7.3 mL/s.), retrograde urethrography (RUG) both dynamic and static, micturating cystourethrography (MCUG), and sonourethrography in some cases.

**Figure 1:**
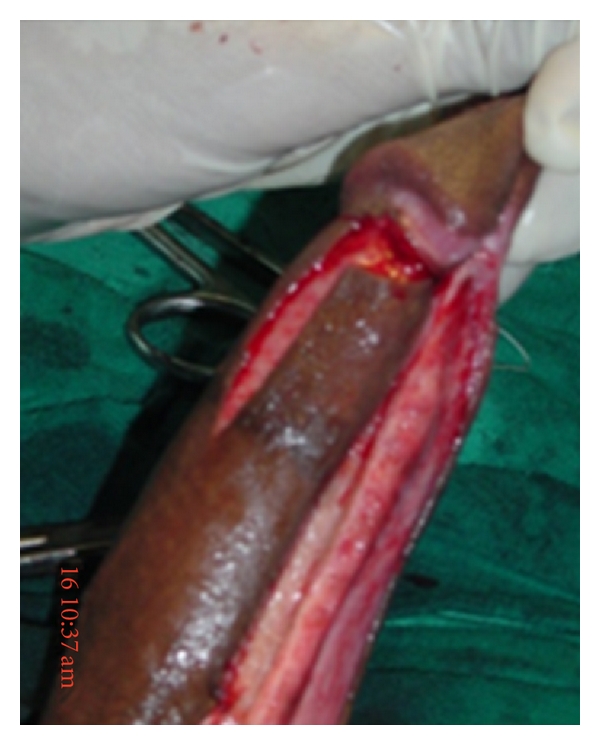
Orandi (longitudinal) penile skin flap.

**Figure 2:**
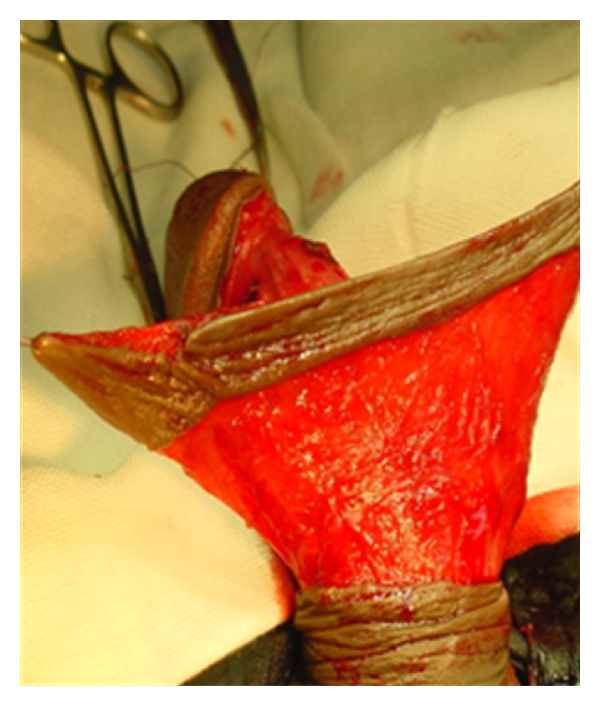
Circular penile skin flap.

**Figure 3:**
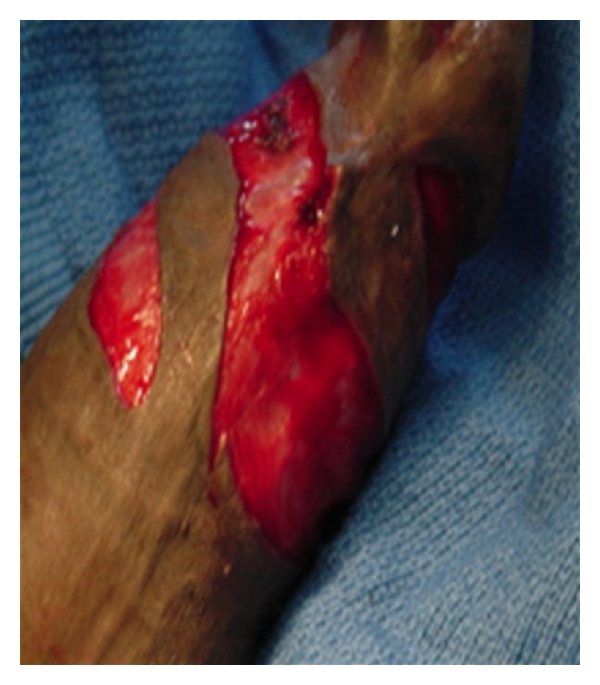
Spiral penile skin flap.

The age of patients was 28–65 years (mean 39.3 year). The etiology of stricture was iatrogenic in 8 cases and post-infective urethritis in 10 cases. The stricture length was 5.6–13.2, located at the penile urethra in 6 cases, bulbar urethra in 2 cases, and combined in 10 cases. All patients were primarily repaired and had ample healthy penile skin with variable flap length, where it was 6 cm in 6 cases subjected to Orandi flap (Figure 1), 7–10 cm in 8 cases with circular fasciocutaneous penile flap (Figure 2), and 10–15 cm in 4 cases with spiral flaps (Figure 3). As regards the technique, the urethra was completely mobilized from the underneath corpora cavernosa and rotated 180 degrees, then, dorsal stricturotomy was done, penile skin flap was applied as a dorsal onlay, and the urethra was fixed to its bed. If penile skin is deficient, U-shaped scrotal flaps were raised for coverage. Indwelling urethral catheter was left for 3 weeks plus suprapubic cystocath. MCUG was performed after catheter removal, if any leakage occurred, cystocath drainage was delayed.

 Followup of all patients after reconstruction included urine flow rate at weekly intervals, RUG at 6–12 weeks, and urethrocystoscopy at 12 and 18 months.

## 3. Results

**Figure 4:**
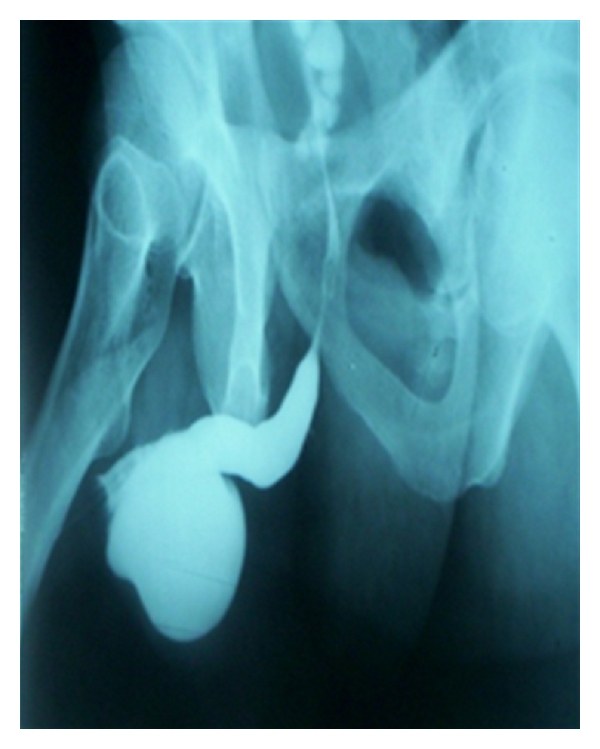
Ascending urethrography Showed a diverticulum in the penile urethra.

 All 18 patients were subjected to flap urethroplasty. The urethra was patent in 14 patients (77%).The mean peak flow rate improved to 22.7 mL/s. The radiographic picture of the reconstructed urethra in RUG was excellent and close to that of a normal urethra in 14 patients in terms of caliber and a smooth transition of the dye into the urethra at both ends. The postoperative complications were as follows: fistula in one patient (5.5%). Restricture occurred in 3 patients (16.6%) and required visual internal urethrotomy. Failure was considered when there was a need for any subsequent urethral procedure as visual internal urethrotomy, urethral dilatation, or urethroplasty. Two patients (11%) showed curvature on erection that does not interfere with sexual intercourse. Diverticulum (in penile urethra) was seen in one patient (5.5%) and it contained stones (Figure 4) and was excised surgically. There was penile skin loss in 3 patients (16.6%). All patients completed at least one-year followup period.

## 4. Discussion

 Penile island flap urethroplasty provides well-vascularized, versatile, and reliable tissue for urethral substitution. It is an ideal procedure for long strictures in the distal urethra [5].

 In our small series of patients, circular [6] (7–10 cm in 8 patients) and spiral flaps [7] (10–15 cm in 4 patients) were fashioned and rotated to be dorsally quilted into the dorsally opened strictured part of the urethra either in penile, bulbar, or bulbopenile uerthra without vascular compromise. The longitudinally fashioned flaps (6 cm in 6 patients) Orandi [8] were comfortably used for penile strictures. The success rate for all penile skin flaps was 77% in our hands approaching that recorded by others [9–11] see Table 1.

 Some authors reported that although there is no reported comparison of dorsally and ventrally quilted onlay flap urethroplasty, in our initial experience dorsal placement was better than ventral placement because (a) the postoperative radiographic studies showed a near-normal restoration of the anatomical appearance of the urethra; (b) there is no sagging, sacculation, and resultant postvoid dribbling, as seen in ventral placements; because the flap sits on the roof of the urethra and is fused with crura (c) the incidence of postoperative fistula is very low. Dorsal placement of the flap is particularly advantageous in the following situations: extension of the flap for meatal and glanular stricture reconstruction and, extension proximally up to the bulbomembranous region and in situations where the reconstruction requests combined flap and free graft [12].

 Most of the complications with skin flap urethroplasty are recurrent stricture, troublesome postvoid dribbling, and diverticulum formation. In previous study, of 17 patients with penile skin urethroplasty, urethral pseudodiverticulum developed in two patients (11.7%). One patient developed a large urethral diverticulum 1 year after procedure and the other 10 years after the procedure [13]. In our study one patient only developed a diverticulum (5.5%) containing stones which was removed surgically.

 In other studies, the stricture recurred in five patients (11%) within a median followup of 27.5 months [12], and of 30 patients with pedicled dorsally quilted penile skin flaps, urethral stricture recurred in 5 (16.6%) [14]. Restricture in our study was encountered in 16.6% (3/18) of patients.

 Any kind of substitution urethroplasty deteriorates over time. Long-term results with skin flap urethroplasty show a decreasing success rate with time [15]. Dorsally quilted penile skin flap urethroplasty seems anatomically more logical; in our hands the early outcome of this technique is encouraging, but we are in need for long-term followup to evaluate the outcomes especially the restructure.

## 5. Conclusion and Recommendations

Free penile skin flaps offer good results (functional and cosmetic) in long penile and/or bulbar urethral strictures. Meticulously fashioned longitudinal, circular, or spiral penile skin flaps could bridge urethral defects up to 15 cm long.

## Figures and Tables

**Table 1:** Flap characters and outcomes of urethroplasty.

Type of skin flap	Length of the flap	Complications
Longitudinal flap (*n* = 6)	6 cm	Urethral patency 14/18 (77%)
Circular flap (*n* = 8 patients)	7–10 cm	Fistulization 1 (5.5%)
Spiral flap (*n* = 4 patients)	10–15 cm	Restricturing 1 (5.5%)
		Curvature on erection 2 (11%)
		Penile skin loss 3* (16.6%)
		Diverticulation 1 (5.5%)

*After completing repair, the penis was covered by U-shaped scrotal flaps.
